# Intercellular adhesion molecule-1 expression in human endometrium: implications for long term progestin only contraception

**DOI:** 10.1186/1477-7827-4-2

**Published:** 2006-01-30

**Authors:** Frederick Schatz, Graciela Krikun, Rebecca N Baergen, Hilary OD Critchley, Edward Kuczynski, Charles J Lockwood

**Affiliations:** 1 Department of Obstetrics, Gynecology and Reproductive Sciences, Yale University School of Medicine, New Haven CT, USA; 2 Department of Pathology, Weil-Cornell Medical Center, New York NY, USA; 3 Centre for Reproductive Biology, University of Edinburgh, UK

## Abstract

**Background:**

Neutrophils infiltrate the endometrium pre-menstrually and after long-term progestin only-contraceptive (LTPOC) treatment. Trafficking of neutrophils involves endothelial cell-expressed intercellular adhesion molecule (ICAM-1). Previous studies observed that ICAM-1 was immunolocalized to the endothelium of endometrial specimens across the menstrual cycle, but disagreed as to whether extra-endothelial cell types express ICAM-1 and whether ICAM-1 expression varies across the menstrual cycle.

**Methods:**

Endometrial biopsies were obtained from women across the menstrual cycle and from those on LTPOC treatment (either Mirena or Norplant). The biopsies were formalin-fixed and paraffin-embedded with subsequent immunohistochemical staining for ICAM-1.

**Results:**

The current study found prominent ICAM-1 staining in the endometrial endothelium that was of equivalent intensity in different blood vessel types irrespective of the steroidal or inflammatory endometrial milieu across the menstrual cycle and during LTPOC therapy. Unlike the endothelial cells, the glands were negative and the stromal cells were weakly positive for ICAM immunostaining.

**Conclusion:**

The results of the current study suggest that altered expression of ICAM-1 by endothelial cells does not account for the influx of neutrophils into the premenstrual and LTPOC-derived endometrium. Such neutrophil infiltration may depend on altered expression of neutrophil chemoattractants.

## Background

The premenstrual human endometrium displays increased prostaglandin-generating capacity, elevated levels of inflammatory cytokines [1,2] and a leukocyte infiltrate that comprises nearly one-half of the cell population [3-5]. Among endometrial leukocyte subtypes, neutrophils are virtually absent until the mid-luteal phase, but comprise a significant portion of the leukocytes in the menstrual phase. During long-term progestin-only contraceptive (LTPOC) administration, the endometrium also experiences enhanced prostaglandin-generating capacity and increased inflammatory cytokine levels [6,7]. Administration of Norplant, which releases levonorgestrel (LNG) from subdermal rods, and Mirena, which releases LNG from an intrauterine system, leads to endometrial infiltration of matrix metalloproteinase-9 (MMP-9) positive neutrophils [8], and macrophages [9], respectively.

Endothelial cell-expressed cellular adhesion molecules mediate leukocyte trafficking [10]. In this regard, particular attention has been directed at the physiological and pathological roles played by intercellular adhesion molecule (ICAM-1), a 76-114-kDa surface glycoprotein that has five extracellular immunoglobulin-like domains [11-14].Transmigration of leukocytes involves high-affinity binding of LFA-1 or Mac-1 on their surface to ICAM-1 expressed on the endothelium [10]. ICAM-1 deficient mice experience numerous inflammatory response abnormalities including impaired neutrophil trafficking [15,16]. Although ICAM-1 has been immunolocalized to the endothelium of various blood vessel types in specimens of cycling endometrium, there are conflicting reports as to whether extra-endothelial cell types also express ICAM-1, and whether ICAM-1 expression varies across the menstrual cycle [17-19]. In view of this lack of consensus, the current study reassessed immunohistochemical (IHC) staining for ICAM-1 in endometrial biopsies across the menstrual cycle, and extended the use of IHC staining of ICAM-1 to include endometrial tissues exposed to subdermal (Norplant) and intra-uterine (Mirena, Schering) exogenous progestogens. Both LTPOC types provide safe and effective contraception for several years. Norplant is particularly well suited for use in underdeveloped countries where access to trained medical personnel is limited. They are discontinued primarily because of inflammation-associated abnormal uterine bleeding (AUB) as a source of personal annoyance and discomfort as well as cultural and religious taboo [20,21]. The levonorgestrel-releasing intra-uterine system (LNG-IUS, Mirena) is now increasingly used as an effective contraceptive and for its associated health benefits, including reduction in menstrual blood loss [22].

Prior to menstruation and during progestin-only contraception (Norplant, Mirena), secretion of MMPs by endometrial leukocytes as well as cytokines that can act as autocrine/paracrine modulators of MMP expression [5], are thought to enhance degradation of the vascular support structure leading to stromal collapse and bleeding [23-26]. The current study sought to determine whether altered expression of ICAM-1 could account for infiltration of neutrophils into the menstrual and LTPOC-derived endometrium.

## Methods

### Tissues

After receiving written informed consent and approval from the Institutional Research Board (IRB) of New York University Medical Center and Bellevue Hospital, specimens of endometrium were obtained across the menstrual cycle (four each from the follicular and luteal phases and five from the menstrual phase) from hysterectomies for benign conditions (e.g. myomas without abnormal uterine bleeding), and histologically dated by the criteria of Noyes et al [27]. For studies on LTPOC-derived endometrium, institutional ethical review and approval was obtained from the New York University IRB and the Lothian Research Ethical Committee, Scotland and written informed consent was obtained for biopsy collection.

The subjects had regular menstrual cycles and had not used hormonal or intrauterine contraception in the six months prior to insertion of Norplant or Mirena. Patients did not exhibit symptoms characteristic of endometriosis such as pelvic pain, dysmennorhea, dysparunia, or infertility. The only way to confirm a diagnosis of endometriosis is through exploratory surgery. Such surgery would be prompted by symptoms that would have ruled out the use of those patients for our study. For the cycling endometrium patients were pre-menopausal between 32 and 43 years of age who were not receiving any hormonal treatments. For the LTPOC endometrium patients were premenopausal, between 28 and 45 years of age, had regular menstrual cycles and had not used any hormonal or intrauterine contraception in the six months prior to receiving the LTPOC treatment.

### Norplant specimens

Prior to insertion of Norplant biopsies were collected from four women (two in the follicular and two in the luteal phase) by Pipelle suction curette (Laboratoire CCD, Paris, France). Only patients who experienced bleeding while on the Norplant treatment were used. Biopsies were collected using an operative hysteroscope connected to a video camera to facilitate separate sampling of bleeding and non-bleeding sites as previously described [25]. These samples were taken after 3 and 12 months post Norplant insertion.

### Mirena specimens

Endometrial biopsies were also obtained from four women (two in the follicular and two in the luteal phase) prior to and at 1, 3, 6, and 12 months after intrauterine insertion of the LNG-intrauterine system by Pipelle suction biopsy.

### Immunohistochemistry (IHC)

Specimens of endometrium obtained across the menstrual cycle as well as from control, and levonorgestrel treated (Norplant, Mirena) subjects were fixed in 4% paraformaldehyde and embedded in paraffin. Four μm sections (4 μm) were deparaffinized, rehydrated and washed in Tris-buffered saline [TBS: 20 mmol/l Tris-HCl, 150 mmol/l NaCl (pH 7.6)], which was used for all washes and for dilution of the antibody. Antigen retrieval was carried-out by incubating sections in sodium citrate buffer (10 mM, pH 6.0) in a microwave oven at 750 Watts for 5 minutes. The sections were then rinsed in 3% hydrogen peroxide to block endogenous peroxidase and incubated for 1 hour at room temperature with either of the following primary antibodies: a goat polycolonal ICAM-1 (CD54) antibody from R&D Systems (R&D Systems, Inc., Minneapolis, MN) or a monoclonal antibody against the Platelet Adhesion Molecule (PECAM) (CD31) from Dako (DakoCytomation California, Inc., Carpinteria, CA). Staining was visualized using the avidin-biotin peroxidase complex (Vectastain ABC kit, Vector Laboratories, Burlingame, CA) and the 3,3'-diaminobenzidine tetrahydrochloride (Sigma-Aldrich, St. Louis, MI) chromogen substrate. Light hematoxylin stain was used for nuclear counterstaining. Negative controls for each tissue section consisted of substituting the corresponding pre-immune serum for the primary antibody.

### Assessment of immunohistochemical (IHC) staining and statistical analysis

Intensity of ICAM-1 staining was evaluated using a semi-quantitative 4-point rating method with the following scoring system: 0, absence of staining; 1, light staining; 2 moderate staining; and 3, strong staining. Each of these possible scores was established in advance of rating the fields via reference to external stained specimens unrelated to this study. In order to determine inter-rater reliability of this scale, two independent judges scored a series of 35 separate fields on slides from 4 separate patient samples. The degree of concordance was then assessed by use of Cohen's kappa statistic, which yielded a value of 0.67, indicating a high degree of agreement between the judges.

Non-parametric statistical analysis was performed by the Mann-Whitney Rank Sum Test with p < 0.05 considered significant.

## Results

**Figure 1:**
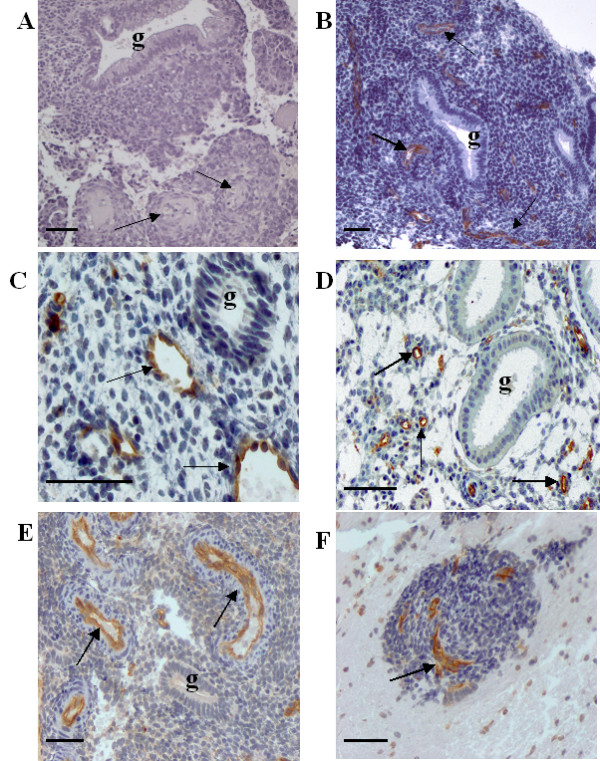
**Immunostaining for ICAM-1 and CD 31 in human endometrium during the menstrual cycle.**Negative control for menstrual endometrium (A). Prominent ICAM-1 staining is evident in the endothelium in endometrial specimens from the follicular phase (C), luteal phase (D), and menstrual phase (E, F). Similar endothelial cell staining intensity and specificity for CD 31 is seen in the menstrual specimen shown in (B). The prominent structure in the menstrual specimen shown in (F) is a "stromal ball," which results from degenerative changes of the stroma. Note the compressed blood vessels displaying prominent immunostaining for ICAM-1, whereas the surrounding stromal cells were only weakly positive. Arrow = blood vessel; g = gland. Bar = 50 μm.

Figure 1 (C-F) displays IHC staining for ICAM-1 in endometrial specimens obtained across the menstrual cycle. As expected, there was a lack of staining in the negative control (A). The endothelium of all specimens examined stained prominently, displaying an intensity that appeared to be independent of hormonal or inflammatory state. Thus, staining intensity did not vary significantly among specimens obtained from the estrogen-regulated follicular phase (C), the progestin-dominated luteal phase (D), or from the pro-inflammatory milieu characteristic of the ovarian steroid withdrawal-initiated menstrual phase (E-F). In both intensity and specificity for the endothelium, immunostaining for ICAM-1 was similar to that of CD31 (shown in Figure 1B), a documented endothelial cell marker whose expression was demonstrated by Tawia [18] to be essentially unchanged across the menstrual cycle. The "stromal ball" displayed by the specimen of menstrual endometrium in (F) indicates that in contrast with the intense IHC staining for ICAM-1 exhibited by the endothelium of the compressed vessels, the stromal cells demonstrate at most only weakly positive immunoreactivity. This observation contradicts a report that stromal cells of cycling human endometrium display significant ICAM-1 IHC staining whose intensity peaks during the menstrual phase [18].

**Figure 2:**
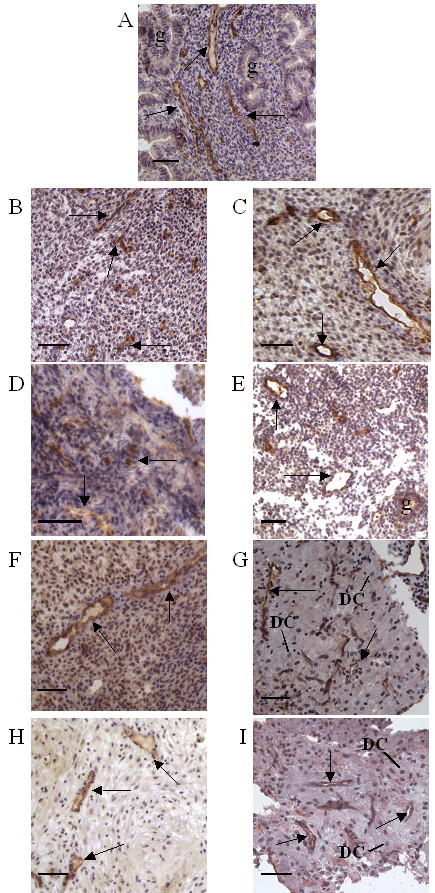
**Immunostaining for ICAM-1 in human endometrium during long term only contraceptive (LTPOC) administration. **IHC staining in the endothelium was prominent and specific for the endothelium in in all endometrial specimens examined. Samples obtained from women using Norplant (subdermal LNG) (B-E)-: (B) 3 months post-Norplant non-bleeding site; (C) 3 months post-Norplant, bleeding site; (D) 12 months post-Norplant, non-bleeding site; and (E) 12 months post Norplant, bleeding site. Similar results were seen in endometrial specimens from women using Mirena (intrauterine LNG) (F-I):-;(F) 3 months post-Mirena, non-bleeding (F); 3 months post-Mirena, bleeding; (G) 12 months post-Mirena, non-bleeding; and (I) 12 months post-Mirena, bleeding. Note that the stromal cells exhibit a much greater decidualization reaction following intrauterine administration of LNG (F-I) than after subdermal LNG (B-E). Arrow = blood vessel; g = gland; DC = decidualized stromal cell. Bar = 50 μm.

Figure 2 displays IHC staining for ICAM-1 in endometrial specimens obtained after administration of the LTPOCs, Norplant (B-E) and Mirena (F-I). As observed in Figure 1 for endometria obtained across the menstrual cycle, Figure 2 demonstrates that imunoreactive ICAM-1 was also localized specifically to the endothelium of LTPOC-derived endometrium with staining intensity that was equivalent among various vessel types and independent of both steroidal and inflammatory state. Thus, consistent with direct delivery of LNG to the uterus with Mirena contraception the progestin-induced decidualization reaction in these specimens (F-I) is much greater than in Norplant-derived specimens (B-E). Moreover, despite the documented increase in pro-inflammatory cytokines [6,7] and leukocyte infiltration [8,9] during LTPOC-induced abnormal uterine bleeding, no differences in endothelial cell IHC staining for ICAM-1 were evident whether the endometrium exhibited abnormal uterine bleeding or gave no indication of bleeding. Specifically, with Norplant administration this comparison was between bleeding (C, E) and non-bleeding sites (B, D) of the same endometrium. With Mirena administration comparisons are between patients experiencing abnormal uterine bleeding G, I) and those who were not bleeding (F, H). For both figures, our rating of staining intensity was characterized by good inter-rater reliability, with a Cohen's kappa value of 0.67 for observations made by two independent observers (See Methods for details).

## Discussion

The current study found that human endometrial endothelial cells displayed prominent IHC staining for ICAM-1 in specimens obtained from the follicular, luteal and menstrual phases, and after administration of the LTPOCs, Norplant (subdermal LNG) and Mirena (intrauterine LNG) and that this staining was of equivalent intensity in all vessels examined. By contrast, the glands exhibited virtually no immunostaining and the stromal cells only weak immunoreactivity. Although IHC staining for ICAM-1 was previously demonstrated in the endometrial endothelium of specimens obtained across the menstrual cycle [17-19], two of the reports found significant ICAM-1 staining in the stromal cells [18,19], with one study noting that ICAM-1 levels in both stromal cells and endothelial cells were elevated in menstrual endometrium compared with specimens examined earlier in the menstrual cycle [18].

The demonstration in the current study that ICAM-1 levels are equivalent in the endometrial endothelium of specimens from the E_2_-dominated follicular phase, the progesterone-exposed luteal phase, and the steroid-withdrawal-initiated menstrual phase suggests that ICAM-1 expression is not under direct ovarian steroid regulation. This conclusion was supported by the ICAM-1 immunostaining results obtained in endometrial biopsies during use of subdermal and intrauterine LNG (Norplant and Mirena respectively) contraception. That both LTPOCs produce a hyperprogestational endometrial milieu is suggested by the observation of significantly high endometrial levels of the progesterone receptor (PR) isoforms PR_A _and PR_B _after administration of Norplant [24] as well as the injectable LTPOC, Provera [23], whereas PR_A _appears to mediate the long-term effects of LNG in the endometrium during intrauterine LNG contraception [6]. Endometrial levels of LNG that are 1000 times greater with intrauterine delivery (Mirena) than with subdermal LNG administration (Norplant) [28]. However, the current study observed no difference in endometrial endothelial ICAM-1 immunostaining regardless of which LNG formulation was evaluated.

Evidence presented in the current study also argues against a role for the local inflammatory milieu in regulating endothelial cell expressed ICAM-1. Thus, equivalent immunostaining intensity was observed when follicular and luteal phase endometria were compared with menstrual, Norplant, and Mirena-derived endometria, which undergo a marked leukocyte infiltration [3-5,8,9] and exhibit other local pro-inflammatory changes such as a high prostaglandin-generating capacity and elevated interleukin-8 (IL-8) levels [1,2,6,7].

ICAM-1 is both constitutively expressed and transcriptionally regulated on the surface of several cell types [29]. Consistent with the latter, the ICAM-1 gene promoter contains several cis-acting elements that predict responsiveness to pro-inflammatory cytokines and reactive oxygen species (ROS). Cooperativity between transcription factors C/EBP and NfκB mediate tumor necrosis factor alpha (TNF-α) and interleukin 1beta (IL-1β) responses. Actions of H2O2 are mediated by antioxidant response elements (ARE), which bind transcription factors AP-1 and Ets [29]. As expected, TNF-α, whose pro-inflammatory activity requires ROS formation, induces ICAM-1 expression in endothelial and epithelial cells and H2O2 induces ICAM-1 expression in endothelial cells. However, H2O2 does not affect ICAM-1 expression in epithelial cells [30].

In vivo studies have generally relied on IHC to localize and assess ICAM-1 levels. Table 1 summarizes the results of nine previous reports, three in nonpregnant endometrium [17-19] and six in pregnant endometrium (decidua) [31-36]. The majority of these agree with the current results, by observing intense ICAM-1 immunostaining in a variety of blood vessel types that appears to be constitutive even in a pro-inflammatory milieu. Recently, in first trimester from cases of hemorrhagic, acutely inflamed, regressing deciduas, we found that endothelial cell ICAM-1 staining intensity was unaffected by the proximity of blood vessels to cytokine-expressing neutrophils [36]. Moreover, despite reports that preeclamptic decidua display a marked macrophage infiltrate and high levels of TNF-α, [37,38] other reports [34,35] found that endothelial cell ICAM-1 expression was similar in decidua from preeclamptic or gestationally age-matched placentas. These studies agree with observations made in the current study for nonpregnant endometrium. It found equivalent ICAM-1 immmunostaining intensity in the endothelium of follicular and luteal phase endometria compared with menstrual, Norplant and Mirena-derived endometria, which exhibit such local pro-inflammatory changes as a marked leukocyte infiltrate [3-5,8,9], high prostaglandin-generating capacity and elevated interleukin-8 (IL-8) levels [1,2,6,7].

**Table 1:** Previously reported ICAM-1 immunostaining in non-pregnant and gestational endometrium. FT: first trimester; FF-PE: formalin-fixed, paraffin-embedded; PE: pre-eclampsia, IUGR: intrauterine growth retardation

**Reference**	**Tissue type**	**Tissue Preparation**	**Endothelial Cell ICAM-1 staining**	**Non-endothelial Cell ICAM-1 staining**
17	Cycling endometrium	Frozen (formalin-fix)	Strong, constitutive	Uniform staining of glands, stroma, and epithelium; strong lymphoid staining
18	Cycling endometrium	Frozen (formalin-fix)	Strong but variable among vessel types with peak at menstrual	Glandular and luminal epithelium negative, stroma weak in proliferative/secretory phases but strong at menstrual
19	Cycling endometrium	Frozen (acetone-fix) and FF-PE	Strong throughout entire cycle	Glandular and luminal epithelium variable, stroma stained throughout cycle with increase expression in menstrual; widespread lymphoid staining
31	FT Decidua	Frozen (acetone-fix)	Strong in all vessel types	Glands negative, stroma weak, moderate staining of lymphocytes
32	FT Decidua, placenta	Frozen (acetone-fix)	Strong in all vessel types	Stroma negative, strong lymphocyte staining, strong staining of decidua parietalis,
33	FT decidua	Frozen (acetone-fix)	Strong in all vessel types	Glands negative, some stroma positive
34	Decidua, placental bed	Frozen (acetone-fix)	Strong, unchanged in normal vs. PE, IUGR, or PE+IUGR	Weak scattered stromal cell staining
35	Decidua, placenta	Frozen (acetone-fix)	Strong, unchanged in normal vs. PE	Villous trophoblasts negative, <10% interstitial trophoblasts stained
36	FT decidua	FF-PE	Strong in all vessel types, same in normal vs. inflammation; constitutive	Glands negative, stroma weak

Regulation of neutrophil migration into inflammatory sites reflects interactions between the IL-8 chemokine and the ICAM-1 adhesion molecule. The former establishes a chemotactic gradient that promotes neutrophil trafficking from the circulation towards the endothelium [39]. This enables the latter to mediate neutrophil rolling and adhesion prior to transendothelial migration [40]. Neutrophils are rich source of gelatinase B (MMP-9) [41], which degrades basement membrane associated collagens IV and V [42]. Moreover, neutrophil-derived MMP-9 cleaves IL-8 to a truncated form [IL-8(7-77)] with 10–30- fold greater potency in promoting neutrophil activation and chemotaxis [41]. The onset of AUB during LTPOC administration stems from fragile, abnormally distended vessels with impaired basement membranes"[43,44]. Administration of LTPOCs produces local hypoxia stemming from reduced uterine vasomotion (45), and increases stromal cell expression of tissue factor, which can generate thrombin at local sites of AUB [46]. The demonstration in the current study that ICAM-1 is constitutively expressed by the endometrial endothelium highlights the important role that altered IL-8 expression plays in regulating neutrophil trafficking into the endometrium. Toward that end, we recently demonstrated that IL-8 expression is enhanced by hypoxia and thrombin in stromal cells derived from pre-decidualized human endometrium [47].

## Conclusion

In the context of our current observations, constitutive endothelial ICAM-1 expression alone cannot account for the marked neutrophil infiltration that characterizes both premenstrual human endometrium as well as the endometrium resulting from LTPOC therapy.

## Authors' contributions

FS designed the study and drafted the manuscript. GK conducted immunohistochemical procedures. RNB assessed immunohistochemical staining. HC performed clinical assessment and collection of specimens related to LTPOC treatment. EK performed the statistical analyses and critically reviewed the manuscript. CJL participated in the conception of the study and critically reviewed the manuscript. All authors read and approved the final manuscript.
